# CO_2_ Uptake and Physicochemical Properties of Carbonation-Cured Ternary Blend Portland Cement–Metakaolin–Limestone Pastes

**DOI:** 10.3390/ma13204656

**Published:** 2020-10-19

**Authors:** Rizwan Hameed, Joonho Seo, Solmoi Park, Issam T. Amr, H.K. Lee

**Affiliations:** 1 Department of Civil and Environmental Engineering, Korea Advanced Institute of Science and Technology, 291 Daehak-ro, Yuseong-gu, Daejeon 34141, Korea; rhameed@kaist.ac.kr (R.H.); junhoo11@kaist.ac.kr (J.S.); 2 Department of Civil Engineering, Pukyong National University, 45 Yongso-ro, Nam-gu, Busan 48513, Korea; solmoi.park@pknu.ac.kr; 3 Carbon Management Division, Research & Development Center, Saudi Aramco, Dhahran 31311, Saudi Arabia; issam.amr@aramco.com

**Keywords:** Portland cement, limestone, metakaolin, carbonation curing, CO_2_ uptake, LC^3^

## Abstract

The feasibility of carbonation curing of ternary blend Portland cement–metakaolin–limestone was investigated. Portland cement was substituted by the combination of metakaolin and limestone at levels of 15%, 30%, and 45% by the mass. The ternary blends were cured with four different combinations of ambient and carbonation curing. The mechanical property, CO_2_ uptake, and mineralogical variations of the ternary blend pastes were investigated by means of compressive strength test, thermogravimetric analysis, and X-ray diffractometry. In addition, volume of permeable voids and sorptivity of the ternary blends were also presented to provide a fundamental idea of the pore characteristics of the blends. The test results showed that the increasing amount of metakaolin and limestone enhanced the CO_2_ uptake, reaching 20.7% for the sample with a 45% cement replacement level at 27 d of carbonation. Meanwhile, the compressive strength of the samples was reduced up to 65% upon excessive incorporation of metakaolin and limestone. The samples with a replacement level of 15% exhibited a comparable strength and volume of permeable voids to those of the sample without substitution, proving that the ternary blend Portland cement–metakaolin–limestone can be a viable option toward the development of eco-friendly binders.

## 1. Introduction

Portland cement (PC) has a global production of more than 4,000 Mt per year, which is 25 times higher than what it was in 1950 [1,2]. The huge production of PC accompanies a significant CO_2_ emission, which accounts for nearly 5–8% of entire CO_2_ emissions [2,3]. Of these emissions, 50–60% are attributed to the calcination of limestone during the manufacturing process of PC, while the other share is from the burning of fossil fuels [1]. Due to the CO_2_ emissions, from the cement industry and other sources, the concentration of CO_2_ in the Earth’s atmosphere has been increased from 280 ppm, in the preindustrial era, to 414.5 ppm in 2020 [4,5]. Different solutions have been adopted to reduce the carbon footprint of the construction industry such as partial replacement of PC with waste materials [6,7,8], alternative clinkers [9], and the use of alkali-activated binders [10,11,12,13]. Recently, the carbonation curing of PC-based materials has become a focus of attention as a potential means of reducing the atmospheric CO_2_ concentration [14,15].

Carbonation curing is the introduction of elevated CO_2_ concentrations into the fresh or premature state concrete [3,15,16]. Although the idea of carbonation curing was first proposed in the 1970s [17,18], it was reluctantly adopted afterwards. However, in recent years amid increasing concerns towards global warming, the interest in the carbonation curing of PC-based materials has been reignited [3,14,15,19,20,21]. In carbonation curing, CO_2_ is exposed to both anhydrates and hydrates. Portlandite is carbonated more significantly as compared to other hydrates till 28 d of carbonation, while the carbonation of C-S-H continues even after 28 d of carbonation. Anhydrous materials carbonate barely in comparison to hydrates [22]. Carbonation curing exhibits certain advantages such as rapid gain in mechanical strength at an early age and enhanced durability [3,15,16]. It is also considered as a potential approach to sequester CO_2_ in PC-based materials [15]. In contrast to conventional steam curing, which requires elevated temperatures of 50–70 °C with higher humidity levels, carbonation curing is less energy-intensive [14]. 

Supplementary cementitious materials (SCMs) are used to reduce the clinker factor of PC due to their lower energy inputs than PC [6,8]. Conventional SCMs have limited amounts to replace the clinkers as global PC alternatives. Slag is available at around 10% of PC production and this proportion is not expected to be increased in the future. Fly ash is produced at around 30% of PC production and is anticipated to be reduced due to the growing environmental concerns related to the coal power generation [6,7,23,24]. On the other hand, clays have a wide availability globally [24]. Metakaolin is formed by dehydroxylation of clays, rich in kaolinite, when calcined at 700–850 °C [24,25,26]. Metakaolin is highly pozzolanic and forms aluminum-containing phases in PC-based systems [24,25,26,27,28]. In addition, carbo-aluminates may also be formed in the presence of freely available carbonates [26]. Limestone is also available worldwide which accelerates the hydration of PC by providing nucleation sites due to additional surface area [29,30]. In addition, the incorporated limestone forms monocarboaluminate and hemicarboaluminate, which helps in the stabilization of ettringite [29].

PC-based systems containing SCMs more than a threshold level, i.e., around 30% of PC, showed declined mechanical performances at an early age [28]. To enhance the early mechanical properties of systems with higher amounts of substitution, an economic solution can be the addition of limestone in the PC-based systems since the limestone provides additional nucleation sites and promotes early hydration [28,31]. Limestone reacts with alumina-containing phases and produce carbonate-AFm phases, yet this reaction pathway is blocked if available alumina is limited in the system [31]. As metakaolin provides a high amount of alumina, combination of limestone and metakaolin can provide better properties even at higher levels of PC substitution [28]. Ternary binder, referred to as limestone calcined clay cements (LC^3^) is gaining attention from the last decade [24,32,33,34,35,36]. LC^3^ can replace PC up to 45% while maintaining comparable performances [28]. Due to the utilization of abundantly available materials in the LC^3^ system, it has the potential to replace PC at a global scale. Pilot production of LC^3^ has been tested in some countries [37,38,39].

Carbonation curing of PC-based materials has been extensively studied during the last decade [3,14,16,19,21]. Studies on the effect of mineral admixtures on carbonation curing suggest that mineral admixtures can enhance CO_2_ uptake [20,40,41,42], particularly a study conducted by Zhang et al. [20] showed that higher levels (50%) of substitution by fly ash can further improve the CO_2_ uptake capacity. To the authors’ knowledge, however, the carbonation curing of the LC^3^ systems has never been tested. This approach can give two-fold benefits, i.e., high substitution of PC and sequestration of CO_2_. With this background, the present research work was aimed at the investigation on the carbonation curing of ternary blends of PC–metakaolin–limestone. Samples of pure PC systems and ternary blends were prepared at the substitution levels of 15%, 30%, and 45% by mass. These samples were tested under four different curing regimes of ambient and carbonation curing. The CO_2_ uptake and physicochemical properties of the blends under various combinations of ambient and carbonation curing were evaluated by compressive strength, X-ray diffraction (XRD), thermogravimetry analysis (TGA), volume of permeable voids, and sorptivity tests.

## 2. Experimental Program

### 2.1. Materials and Sample Preparation

Portland cement (PC), conforming to the ASTM C150, was obtained from Sungshin Cement Co., Ltd., South Korea. Metakaolin, branded as MKC100, was supplied by Nycon Materials Co., Ltd., South Korea, and commercially available limestone powder was procured from Duksan Reagents Company, Co., Ltd., South Korea. The chemical composition determined by the qualitative X-ray fluorescence (XRF) analysis is presented in Table 1. Note that the loss-on-ignition was not tabulated here since Table 1 shows qualitative XRF results, yet it is anticipated that the limestone might exhibit 40–50 wt % of loss-on-ignition due to the presence of carbonates. Basic properties of PC used in this study are presented in Table 2.

Mix proportion of the samples are shown in Table 3. Four different mixtures were prepared: one with PC only, while the other three mixtures had different replacement levels of PC by the combination of metakaolin and limestone. In these mixtures, the weight ratio of metakaolin and limestone powder was kept as 2:1 based on previous studies [28,31,32,35]. PC was replaced with the combinations of metakaolin and limestone at varying weight percentages of 15%, 30%, and 45%. The sample ID was designated based on the replacement levels of PC, for instance, ML15 indicates a mixture replacing PC with 10 wt % of metakaolin and 5 wt % of limestone. Paste samples with a constant water-to-binder ratio of 0.5 were fabricated. Dry materials were mixed for 3 min. After dry mixing, water was added and further mixed for 5 min in order to make uniform and homogenized pastes. Fresh slurry was poured into 50-mm cubes, 40 × 40 × 160 mm prisms, and Φ100 × 50 mm cylinders for compressive strength tests, carbonation degree measurement, and durability tests including volume of permeable voids and sorptivity, respectively. All samples were sealed with plastic wraps immediately after casting to avoid the evaporation of water.

### 2.2. Curing Conditions and Test Methods

**Figure 1:**
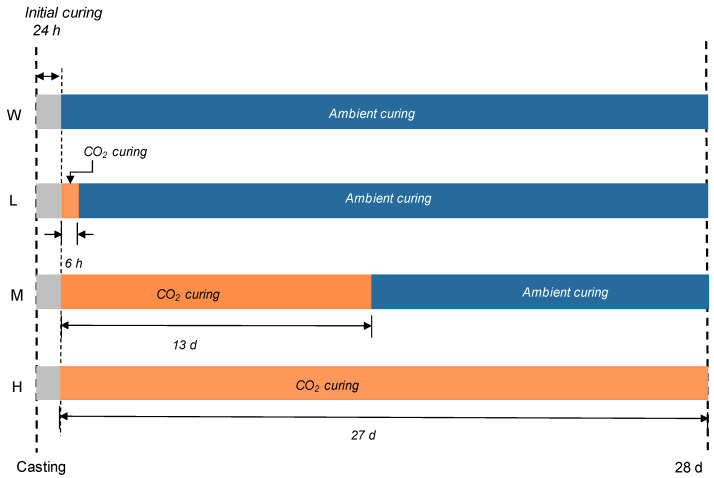
Curing regimes used in this study.

The curing regimes used in this study are summarized in Figure 1. All samples were commonly allowed 24 h of initial curing at 20 °C. After initial curing, the samples underwent four different curing regimes. W-series samples were cured at ambient conditions for 28 d. L-, M-, and H-series samples were carbonation-cured for 6 h, 13 d, and 27 d, respectively. After carbonation curing, L- and M-series samples were cured at ambient conditions until 28 d. Ambient conditions here describe the sealed curing of the samples at 23 ± 2 °C. For carbonation curing, CO_2_ concentration, temperature, and relative humidity were 10%, 20 °C, and 60%, respectively. Samples for chemical analyses were crushed and sieved by a 3 mm sieve before carbonation in order to get a uniform carbonation regardless of location. It should be noted here that complete nomenclature of the samples includes curing condition, for instance, ML30-M indicates a set of samples with a mixture of ML30 (70% PC, 20% metakaolin, and 10% limestone) with the curing regime following M-series.

The compressive strength of samples at 28 d of curing was determined in accordance with ASTM C109 [43] by using a 250 kN universal testing machine at a loading rate of 0.5 MPa/s. The representative strength value was averaged from three replicas. The carbonation degree of the samples was measured by spraying a 1% phenolphthalein indicator onto the cross-section of the carbonated prisms [19,44]. The carbonation degree for the L-, M-, and H-series samples was determined immediately after carbonation curing, i.e., after 6 h, 13 d, and 27 d of carbonation for L-, M-, and H-series samples, respectively. In addition, carbonation degree of the samples at 3 and 7 d of carbonation was additionally provided to observe the progressive carbonation degree. The carbonation degree was determined by Equation (1) [19,44]. The pH value of pore solution in the samples was measured using a suspension made with powdered sample and deionized water. A 2 g measure of a sample was immersed in 10 mL of deionized water and stirred at 200 rpm for 10 min before pH measurement.

(1) $$\mathrm{Carbonation}\;\mathrm{degree}\;(\%)\;=\frac{\mathrm{Carbonated}\;\mathrm{area}\;}{\mathrm{Total}\;\mathrm{cross}-\mathrm{sectional}\;\mathrm{area}}\;\times \;100$$

Characterization of mineral phases was carried out by means of XRD analysis at 28 d of curing. The XRD was performed using an Empyrean instrument under a CuKα radiation with current and voltage of 30 mA and 40 kV, respectively. The XRD patterns were collected in a 2θ° range of 5–65 2θ° with a step size of 0.026 2θ° and a step time of 1.58s. Thermal evaluation of hydrates present in the samples was done by TGA at 28 d of curing. The weight variation of the samples was monitored in the temperature range of 25–1000 °C with a fixed heating rate of 10 °C/min. N_2_ gas was constantly injected during the measurement so as to avoid oxidization of the samples. CO_2_ uptake was measured from TGA curves. Percentage mass loss was quantified by the tangential method to calculate the mass loss associated with the decarbonation of CaCO_3_ [36,45].

Sorptivity test for all samples was performed at 28 d of curing, in accordance with the ASTM C1585 [46]. The initial mass of the samples was determined after sealing the side surfaces. Then, these samples were immersed in 3 mm deep water. Mass of the samples in surface dry condition was frequently measured at time intervals specified in ASTM C1585. The absorption (I) was measured following Equation (2) [46].

(2) $$I\;=\frac{m_{t}}{a\;\times \;d}$$

where *m_t_*, *a*, and *d* denote variation of sample mass (g), sample area exposed to water (mm^2^), and density of water (g/mm^3^), respectively. Initial and final sorptivity coefficients were determined as the slope of the best fit line between *I* and the square root of time (s^0.5^), from 1 min to 6 h, and 1 d to 7 d, respectively. Volume of permeable voids of the samples were tested at 28 d of curing, in accordance with the ASTM C642 [47].

## 3. Results and Discussion

### 3.1. Compressive Strength

**Figure 2:**
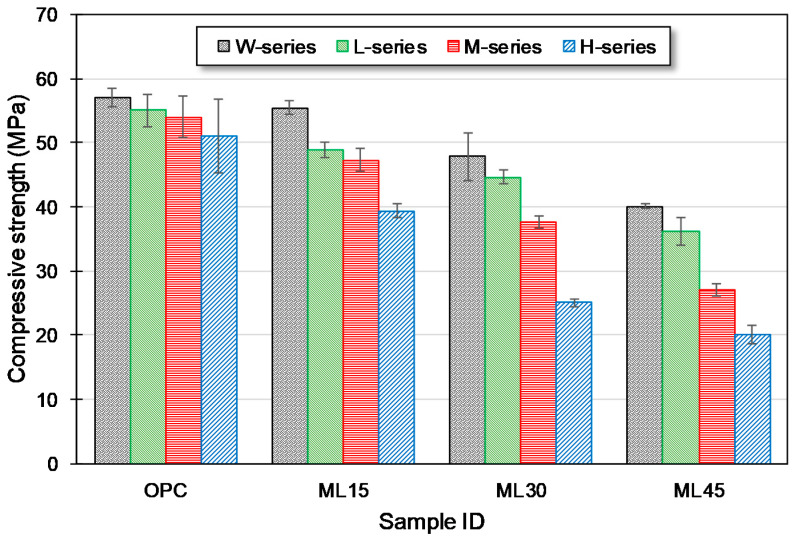
Compressive strength of samples at 28 d of curing.

The compressive strength of the samples after 28 d of curing is shown in Figure 2. The compressive strength of the W-series samples decreased as PC replacement level increased. Comparable compressive strength to the OPC-W sample was observed in the ML15-W sample. This agrees with the outcomes reported in a previous research [31]. While with the higher PC replacement level, a declined mechanical behavior was observed in comparison with the OPC-W sample. The ML30-W and ML45-W samples exhibited compressive strengths of 47.8 and 40.1 MPa, respectively. Previous studies of PC-metakaolin-limestone blends presented identical results to what reported in the present study [28,31]. The OPC samples exhibited comparable compressive strength for all curing regimes, i.e., ambient and carbonation curing regimes. Chen et al. [48] described that carbonation-cured PC-systems show improvement in compressive strengths at early ages while the positive effect weakens with longer age. Other than this, the optimal pre-curing duration before start of carbonation curing, also depends with the carbonation duration; it decreases with the increase in carbonation curing duration [48]. Blended samples showed more prominent behavior with an increase in carbonation duration. L-series samples showed comparable compressive strengths with their W-series counterparts. The duration of carbonation affected the mechanical strength. Among blended samples, compressive strength of the ML15 samples was observed to be comparable for the L- and M-series samples, but showed a notable reduction in strength of the H-series sample. The ML30 and ML45 samples showed declined compressive strengths upon an increment in the carbonation durations. The reduction in the compressive strength of the blended samples upon carbonation can be attributed to their increased overall porosity (explained in Section 3.5) and reduced amount of portlandite upon higher replacement of PC [42]. Zhang et al. [20] described that pozzolanic reaction is hindered by the early carbonation curing. Carbonation reaction reduces the alkalinity by consuming portlandite which is essential for pozzolanic reaction. This effect is more prominent with an increase in the carbonation time and higher substitution levels. Due to this hindrance in pozzolanic reaction, most portion of the SCM acts as a filler material in the paste which might be the reason for lower compressive strengths of blended pastes for longer carbonation durations.

### 3.2. Carbonation Degree and pH Variation

**Figure 3:**
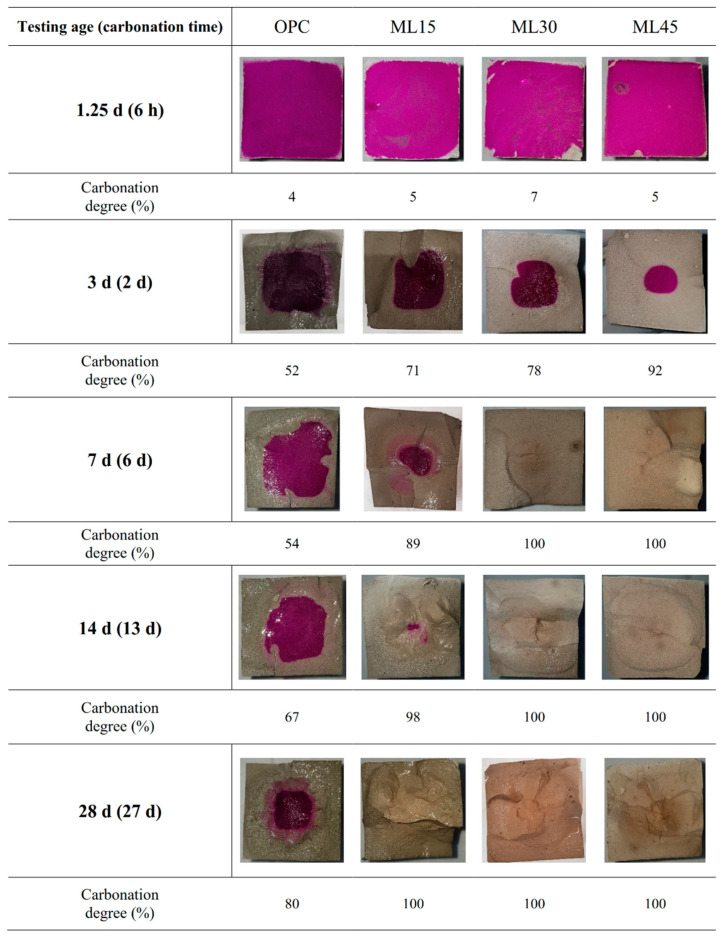
Carbonation degree of the samples.

Carbonation degree of the samples is shown in Figure 3. After 6 h of carbonation curing, all samples showed almost similar extent of carbonation degree, i.e., 4–7%. With an increase in the carbonation duration, samples exhibited different aspect of carbonation degree. The OPC samples showed a remarkable increase in the carbonation degree from 6 h to 2 d of carbonation. The carbonation degree of the OPC samples kept increasing at a steady rate until 27 d of carbonation. At 27 d of carbonation curing, 80% of the cross-sectional area of the OPC samples was carbonated. The carbonation degrees of the OPC samples were similar to those reported in previous works [19,44]. Blended samples with limestone and metakaolin showed much higher rates of carbonation degree than OPC samples at all ages. For the ML15 samples, the carbonation degree reached a value of 71% and 89% at 2 d and 6 d of carbonation and almost completely carbonated at 13 d of carbonation. With the increase in the replacement level of PC, the extent of carbonation also surged. The carbonation degree of ML45 samples was 92% even at 2 d of carbonation curing. At 6 d of carbonation curing, both ML30 and ML45 samples were fully carbonated.

**Figure 4:**
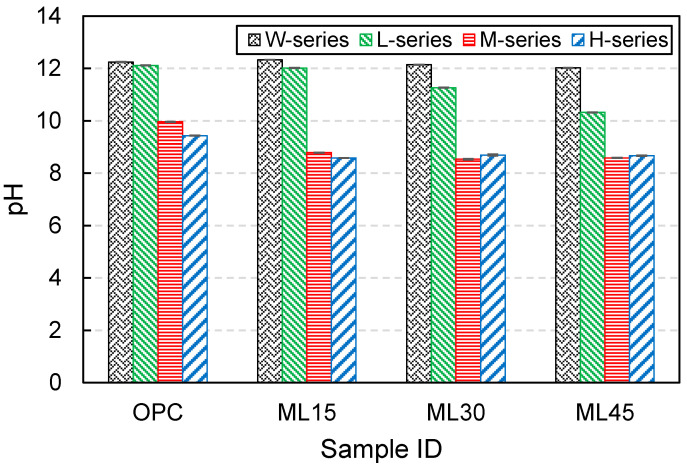
pH value of samples at 28 d of curing.

The pH value of the samples at 28 d of curing is shown in Figure 4. The pH value of the samples cured with the W-series regime was similar regardless of the substitution level. Only a slight reduction in the pH value of W-series samples was observed with an increase of substitution level. This trend became clear in the L-series samples due to the dilution effect associated with the substitution of PC with metakaolin and limestone. The M-series samples showed a notable reduction in the pH values, among which the OPC sample maintained pH value approximately at 10. The blended M-series samples were found to be almost fully carbonated as the pH value of them reached 8. This was reflected in the carbonation degree of the samples at 13 d of carbonation (see Figure 3). For H-series samples, all but OPC sample exhibited similar pH level, meaning the entire carbonation of the samples.

### 3.3. Phase Identification by X-Ray Diffractometry

**Figure 5:**
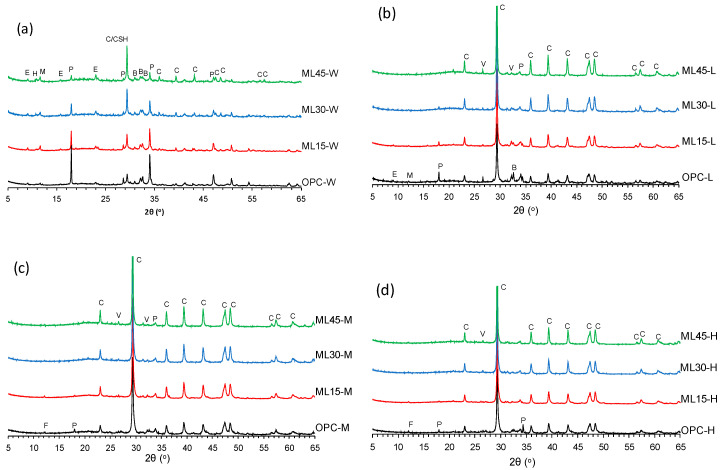
XRD patterns of (**a**) W-, (**b**) L-, (**c**) M-, and (**d**) H-series samples. The annotations are as follows: B—belite, C—calcite, CSH—calcium silicate hydrate, E—ettringite, F—brownmillerite, H—hemicarboaluminate, L—larnite, M—monocarboaluminate, P—portlandite, and V—vaterite.

The XRD patterns of W-series samples are presented in Figure 5a. The OPC-W sample showed peaks related to the presence of portlandite, calcite, C-S-H, and ettringite. Among the blended samples, the ML15-W sample showed portlandite peaks with the highest intensity which were still lesser than that of OPC-W peaks. The intensity of portlandite peaks got reduced for higher substitution levels of PC. Previous studies show that the reaction of metakaolin and limestone in blended systems consumes portlandite and overall reduction in the amount of PC in these blends also contributes in reduction of portlandite formation [28,31]. Peaks associated with two AFm phases—i.e., hemicarboaluminte and monocarboaluminate—were also observed at 10.7 2θ° and 11.6 2θ°, respectively, agreeing with available literatures [28,31,33]. The intensity of these phases was increased with higher replacement levels of PC in the blended samples [31]. Peaks related to ettringite were also present in the blended samples. Small peaks associated with the unreacted belite were also observed in the W-series samples.

The XRD patterns of L-, M-, and H-series samples are shown in Figure 5b–d, respectively. All samples showed strong peaks related to calcite. Intensity of portlandite peaks was observed to be decreased for the OPC-L sample in comparison to the OPC-W sample, which further decreased in the OPC-M and OPC-H samples. Reduction in portlandite peak intensity verifies the conversion of portlandite to calcite due to the carbonation curing [42]. For blended samples, portlandite peaks were observed to be reduced in intensity with increase in the carbonation duration. This reduction was also proportional with the increase in substitution levels; samples with high substitution levels—i.e., ML30 and ML45 samples—mainly showed peaks related to calcium carbonate polymorphs. Peaks related to AFm and AFt phases also vanished with the carbonation curing. Carbonation-induced decomposition of these phases is evident from previous research works [48]. M- and H-seires samples also showed peaks related to brownmillerite, whose inetnsity also reduced with increased replacement levels of PC.

### 3.4. CO_2_ Uptake by Thermogravimetric Analysis

**Figure 6:**
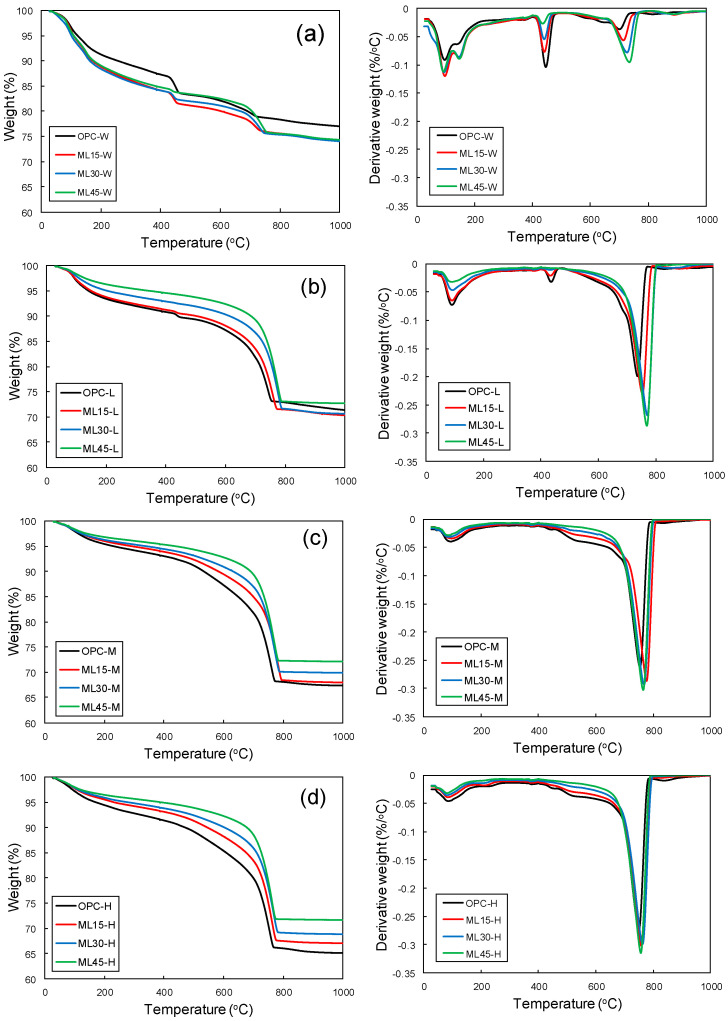
Thermogravimetry analysis curves of (**a**) W-, (**b**) L-, (**c**) M-, and (**d**) H-series samples.

TGA curves of the samples are shown in Figure 6. The W-series samples showed weight loss humps at around 100 °C and a shoulder around 140 °C due to the dehydration of chemically attached water from C-S-H, ettringite, and AFm phases [16,44,49,50]. It is reported that the weight loss hump at around 140–160 °C is mainly associated with the presence of monocarboaluminates and hemicarboalumiates [28]. Weight loss hump observed from 420 °C to 500 °C showed the dehydroxylation of portlandite [16,51]. The OPC-W sample showed the highest amount of portlandite. In the blended samples, a reduction of portalndite was observed; with higher substitution levels, higher consumption of portlandite was observed, which can also be seen in XRD results. Weight loss humps in the temperature range of 550–800 °C can be attributed to the decarbonation of CaCO_3_ [52,53].

The L-, M-, and H-series samples exhibited a reduced weight loss related to dehydration of water from C-S-H, ettringite, and AFm phases (Figure 6b–d); more reduction was observed at increased carbonation curing durations. The weight loss hump in the temperature range of 420–500 °C, associated with the presence of portlandite, disappeared for all the samples except for the OPC-L and ML15-L samples. For the M- and H-series samples, no weight loss humps were observed for portlandite, reflecting the complete consumption of portlandite by carbonation. All L-, M-, and H-series samples showed decarbonation of calcite with strong weight loss humps. CO_2_ uptake for the carbonated samples is presented in Table 4. For the blends with limestone powder, percentage mass loss originated from the limestone was eliminated to genuinely identify the quantity of carbonation products. The OPC samples showed a similar carbonation uptake to those reported in the literature [19]. It was observed that the CO_2_ uptake capacities of the samples have strong relation with carbonation-curing duration and PC replacement levels: highest CO_2_ uptake capacity was observed for ML45-H samples. Tu et al. [54] explained that the increase in carbonation capacity of systems with limestone are due to two physical effects: dilution and nucleation. In dilution effect, the cement particles are more sparsely spread and, as a result, CO_2_ access to particles become easier, while limestone powder in a system provide more nucleation sites on which carbonation products can precipitate. It is also reported that limestone shows higher affinity for carbonation products (CaCO_3_) due to higher molecular recognition and improves the CO_2_ uptake [54].

### 3.5. Volume of Permeable Voids and Sorptivity

The volume of permeable voids and sorptivity coefficients of the samples under various curing regimes are shown in Table 5. The OPC samples showed a slight reduction in the volume of permeable voids with the increase in carbonation curing duration. The ML15-W samples exhibited almost similar volume of permeable voids as that of OPC-W samples, while ML30-W and ML45-W samples showed higher values of volume of permeable voids. Previous research works also described that the total porosity of blended systems was higher than that of pure PC systems and only blends with up to 15% replacement of PC by combined substitution by limestone and metakaolin exhibited similar porosity to that of PC systems [28,31]. Among carbonated blended systems, ML15 samples showed a slight reduction in the volume of permeable voids, while the ML30 and ML45 samples presented increase in the volume of permeable voids with the increase of carbonation curing duration. Initial and secondary sorptivity coefficients also explained the reduction in the volume of permeable voids for the OPC samples, while slight reduction can be observed for ML15 samples. In contrast, increase in sorptivity coeficients was observed for ML30 and ML45 samples with increase in carboantion curing durations. Qin et al. [42] reported an increase in the total porosity of blended systems upon carbonation curing. In general, carbonation of portlandite reduces the porosity of PC-based systems, but in systems where portlandite quantity is low due to pozzolanic reaction and reduced clinker content, carbonation of C-S-H takes place which coarsens the porosity [53]. The longer duration of carbonation exhibited more carbonation of the C-S-H phase which reflects the porosity results.

## 4. Conclusions

The present study investigated the effect of carbonation curing on the PC-metakaolin-limestone ternary blends. Ternary blends replacing the PC with the combinations of metakaolin and limestone by mass levels of 15%, 30%, and 45% were exposed to four different combinations of ambient and carbonation curing regimes. Performances of these blends were evaluated by means of compressive strength, carbonation degree, XRD, TGA, volume of permeable voids, and sorptivity tests. Key findings obtained from the study are summarized below:

(1) The compressive strength of the blended samples exhibited a reduction in the strength as compared with that of the OPC sample. The loss of the compressive strength was increased as the substitution level increased from 15% to 45%. In addition, an increase in the duration of carbonation from 6 h to 27 d resulted in the significant loss of strength levels. The blends with a high substitution and longer exposure duration to CO_2_ experienced significant changes in mechanical strength.
(2) Blended samples showed higher rates of carbonation than the OPC samples at all carbonation curing ages. At 27 d of carbonation curing, OPC sample showed carbonation degree of 80%, while the ML30 and ML45 samples exhibited complete carbonation even at 6 d of carbonation. Carbonation degree was governed both by carbonation duration and by cement replacement level.
(3) The XRD and TGA analyses showed the consumption of portlandite upon carbonation, which was proportional with the carbonation-curing duration. Upon carbonation, the main phases observed were CaCO_3_ polymorphs.
(4) The replacement of the PC by metakaolin and limestone vastly improved the CO_2_ uptake capacity, showing environmental benefits. The increase in the CO_2_ uptake of the ML45 samples with respect to the OPC samples was 54%, 42%, and 50% for L-, M-, and H-series, respectively.
(5) An increase in the volume of permeable voids was observed upon exposure to CO_2_ for the blended samples due to reduced portlandite amount which promoted carbonation of C-S-H which ultimately coarsens the porosity. The ML45-H sample showed volume of permeable voids of 38.6% which is 18.4% higher than that of the OPC-W sample.

## Figures and Tables

**Table 1:** Chemical composition of the raw materials used in this study.

wt %	Portland Cement	Metakaolin	Limestone
CaO	62.50	0.92	99.20
SiO_2_	21.00	50.10	0.08
Al_2_O_3_	5.90	38.40	0.01
Fe_2_O_3_	3.20	5.69	0.03
MgO	0.11	0.11	0.28
R_2_O	0.80	0.62	0.01
SO_3_	2.10	0.05	0.01
TiO_2_	0.38	3.45	-
P_2_O_3_	0.14	0.09	0.01
Mn_2_O_5_	0.10	0.01	-
SrO	0.15	0.06	0.23

**Table 2:** Properties of Portland cement provided by manufacturer.

Portland Cement	
Fineness	3450 cm^2^/g
Initial setting time	225 min
Final setting time	345 min
Density	3.14 g/cm^2^
Standard compressive strength development	
3 day	15.6 MPa
7 day	25.2 MPa
28 day	51.2 MPa

**Table 3:** Mixture proportion of the samples expressed as mass ratio.

Sample ID	Portland Cement	Metakaolin	Limestone	Water/Powder ^1^ Ratio
OPC	1	0.00	0.00	0.5
ML15	0.85	0.10	0.05	0.5
ML30	0.70	0.20	0.10	0.5
ML45	0.55	0.30	0.15	0.5

^1^ Powder denotes the summation of Portland cement, metakaolin, and limestone.

**Table 4:** CO_2_ uptake capacity of the carbonated samples.

Sample ID	CO_2_ Uptake (g/100g of Powder ^1^)		
	L-Series	M-Series	H-Series
OPC	11.3	13.7	13.8
ML15	16.4	19.1	20.1
ML30	16.6	19.3	20.1
ML45	17.4	19.4	20.7

^1^ Powder denotes the summation of Portland cement, metakaolin, and limestone.

**Table 5:** Volume of permeable voids and sorptivity coefficients of the samples under various curing regimes.

Sample ID	Volume of Permeable Voids (%)	Initial Sorptivity Coefficient (×10^−3^ mm/s^1/2^)	Secondary Sorptivity Coefficient (×10^−3^ mm/s^1/2^)
OPC-W	32.6	9.5	0.12
ML15-W	33.7	8.8	0.11
ML30-W	34.8	6.5	0.13
ML45-W	35.2	6.1	0.14
OPC-L	30.4	8.3	0.10
ML15-L	32.1	7.9	0.11
ML30-L	35.9	8.5	0.13
ML45-L	36.3	9.4	0.15
OPC-M	27.4	7.3	0.10
ML15-M	31.0	7.8	0.13
ML30-M	34.6	11.1	0.17
ML45-M	38.6	12.3	0.18
OPC-H	24.9	6.8	0.11
ML15-H	30.4	7.7	0.13
ML30-H	35.1	10.6	0.17
ML45-H	38.6	11.8	0.18
